# Acetate Induces Growth Arrest in Colon Cancer Cells Through Modulation of Mitochondrial Function

**DOI:** 10.3389/fnut.2021.588466

**Published:** 2021-04-15

**Authors:** Meliz Sahuri-Arisoylu, Rhys R. Mould, Noriko Shinjyo, S. W. Annie Bligh, Alistair V. W. Nunn, Geoffrey W. Guy, Elizabeth Louise Thomas, Jimmy D. Bell

**Affiliations:** ^1^Research Centre of Optimal Health, School of Life Sciences, University of Westminster, London, United Kingdom; ^2^Health Innovation Ecosystem, University of Westminster, London, United Kingdom; ^3^School of Health Sciences, Caritas Institute of Higher Education, Hong Kong, China

**Keywords:** acetate, short chain fatty acid, mitochondria, ROS, Warburg effect

## Abstract

Acetate is one of the main short chain fatty acids produced in the colon when fermentable carbohydrates are digested. It has been shown to affect normal metabolism, modulating mitochondrial function, and fatty acid oxidation. Currently, there is no clear consensus regarding the effects of acetate on tumorigenesis and cancer metabolism. Here, we investigate the metabolic effects of acetate on colon cancer. HT29 and HCT116 colon cancer cell lines were treated with acetate and its effect on mitochondrial proliferation, reactive oxygen species, density, permeability transition pore, cellular bioenergetics, gene expression of acetyl-CoA synthetase 1 (*ACSS1*) and 2 (*ACSS2*), and lipid levels were investigated. Acetate was found to reduce proliferation of both cell lines under normoxia as well as reducing glycolysis; it was also found to increase both oxygen consumption and ROS levels. Cell death observed was independent of *ACSS1/2* expression. Under hypoxic conditions, reduced proliferation was maintained in the HT29 cell line but no longer observed in the HCT116 cell line. *ACSS2* expression together with cellular lipid levels was increased in both cell lines under hypoxia which may partly protect cells from the anti-proliferative effects of reversed Warburg effect caused by acetate. The findings from this study suggest that effect of acetate on proliferation is a consequence of its impact on mitochondrial metabolism and during normoxia is independent of *ACCS1/2* expression.

## Introduction

Acetate, one of the main short chain fatty acids produced as a result of ingestion of fermentable carbohydrates has previously been shown to demonstrate anti-tumorigenic effects following its delivery as acetate encapsulated in liposomes (1). However, there is conflicting evidence regarding its effects; acetate has been shown to reduce cell viability of colon cancer cells *in vitro* (2, 3) and reduce cancer cell proliferation in the liver *in vivo* (4). Moreover, acetate has also been shown to increase colon cancer cell death in combination with propionate; an effect further increased at a reduced pH (5). Conversely, others have reported no effect of acetate on colon cancer cell lines, despite showing that other short chain fatty acids, butyrate and propionate, reduce cell proliferation (6–9). Although the underlying mechanisms have not been fully elucidated, Jan et al. suggest that acetate (and other SCFAs) induce cell death through mitochondrial changes, such as swelling and increased ROS production (3).

Recent findings have suggested that there may be different mechanisms which account for the effect of acetate on cancer cell proliferation. Long et al. have shown that glyceryl acetate (GTA), an FDA approved food additive, is hydrolyzed in cells to produce free acetate, reducing the growth of malignant brain tumors through cell growth arrest (10). Whereas, Mashimo et al. have reported that acetate is oxidized by tumor cancer cells and increases cytosolic acetyl-CoA synthetase (*ACSS2*) expression, making tumors more aggressive as silencing these genes is known to reduce malignancy (11). Indeed, Comerford et al. and Schug et al. have shown that acetate is vital for tumor growth since silencing the *ACSS2* gene suppresses tumor growth in both liver (12) and prostate cancer cell lines (13).

We have previously reported that acetate has beneficial effects on mitochondria of liver and adipose tissue, improving oxidative phosphorylation (14). As initially observed by Warburg, cancer cells under normoxia convert glucose to lactate and have reduced oxidative phosphorylation and increased glycolysis (15). We have previously shown reduced tumor growth with acetate delivery to tumors *in vivo* (1), here we investigate the metabolic effects of acetate on colon cancer cell lines HT29 and HCT116 as a possible mechanism for reduced tumor growth.

## Materials and Methods

### Cell Culture

HT29 and HCT116 colon cancer cell lines were obtained from ATCC (LGC Standards, Middlesex, UK) and grown in DMEM (Sigma-Aldrich, UK) and RPMI (ThermoFisher Scientific, USA) media, respectively, supplemented with 1% glutamate (ThermoFisher Scientific, USA) and penicillin and streptomycin (Sigma-Aldrich, UK) and 10% FBS (ThermoFisher Scientific, USA) at 37°C with 5% CO_2_. Cells were used for experimental procedures between passage numbers 3 and 18.

### Cell Growth and Proliferation

For cell growth, cells were seeded in 96-well plates at 20 × 10^3^ cells per well and incubated for 24 h, followed by 24 h treatment with 1 or 10 mM sodium acetate (NaAc). One and 10 mM NaAc were diluted in media from a 1 M stock solution which was prepared by dissolving sodium acetate salt (Sigma-Aldrich, UK) in deionised water, adjusting pH to 7.4 by NaOH and filtering. Cell growth was measured using sulforhodamine B (SRB, Sigma-Aldrich, UK) colorimetric assay as described previously (16). Briefly cells were fixed with 10% (w/v) trichloroacetic acetic acid solution at 4°C for 1 h, rinsed, and stained by 0.4% (w/v) SRB in 0.1% (v/v) acetic acid for 30 min. The plates were washed by 0.1% (v/v) acetic acid to remove unbound SRB dye, and the dye was dissolved by 10 mM Tris base. Absorbance was measured at 500 nm and at 690 nm (as background), using a microplate reader (SPECTROStar Nano, BMG LABTECH, Ortenberg, Germany).

For cell proliferation, cells were seeded 20 × 10^3^ cells per well on a 96-well plate and treated with 10 mM NaAc for 24 h. On the day of the assessment, cells were treated with BrdU, fixed and assay was carried out using a BrdU Cell Proliferation Assay (Millipore) according to the manufacturer's instructions. Plates were read using a SPECTROStar Nano (BMG Labtech, Germany) at 450 and 540 nm.

ATP/ADP ratio was measured to assess the effects of acetate on apoptosis and necrosis. Cells were seeded 20 × 10^3^ cells per well on 96-well plates. On the following day, cells were treated with control, 1 or 10 mM NaAc for 24 h. ATP and ADP levels (*n* = 4) were measured using ADP/ATP Ratio Assay Kit (Merc, UK) by FLUOstar Optima (BMG Labtech, Germany).

### ROS Assay

For detection of cellular ROS, cells were seeded 25 × 10^3^ cells per well on a 96-well plate and received either control, 1 or 10 mM NaAc treatment for 15 min or 24 h. Then cells were stained with 2′,7′-dichlorofluorescin diacetate (DCFDA) using DCFDA Cellular ROS Detection Kit according to the manufacturer's instructions (Abcam, USA) and read on a fluorescent plate reader (ex/em: 495/529 nm) (FLUOstar Optima).

### Citrate Synthase (CS) Activity Assay

To assess mitochondrial density CS activity assay was employed. Cells were seeded 6 × 10^6^ per flask and next day treated with control, 1 or 10 mM acetate for 24 h (*n* = 3). Cells were then collected, and CS activity was measured with Citrate Synthase Activity Assay Kit (Merc, UK) according to manufacturer's instructions and read for 40 min with 5 min intervals at 412 nm using SPECTROStar Nano.

### Ca^2+^ Retention Capacity (CRC) Assay

To assess the opening of mitochondrial permeability transition pore (mPTP), a CRC assay was carried out. Cells were seeded 6 × 10^6^ per flask and next day treated with control, 1 or 10 mM acetate for 24 h (*n* = 3). Cells were then collected and mitochondria isolated using a Mitochondria Isolation Kit (Abcam, UK). Protein concentration of mitochondria were measured using a Bradford protein assay (Bio-Rad, US). Isolated mitochondria were washed with and reconstituted in mitochondrial assay buffer (MAB) at 2 mg/ml concentration and incubated with 0.7 μM Fluo-4FF (ThermoFisher Scientific, USA) prepared in MAB supplemented with 20 mM glutamate (Merc, UK) and 4 mM malate (Merc, UK) for 10 min in black 96-well plates. After a basal measurement, 100 μM CaCl_2_ (Merc, UK) was added into each well, this was repeated for 10 times, with 3 min between each dose. Repeated measurements were made in between injections (17) using FLUOstar Optima (ex./em.: 485/570 nm). Data were analyzed as change from minimum and maximum fluorescence values. Area under the CRC curves were also calculated.

### Cellular Bioenergetics

Oxygen consumption rate (OCR) and extracellular acidification rate (ECAR) was measured using a XFe24 Extracellular Flux Analyzer (Seahorse Bioscience) and the experiments were performed according to the manufacturer's protocols using 20 × 10^3^ HT29 and 30 × 10^3^ HCT116 cells/well in 24-well plates. For 12 h continuous measurement, cells were treated with acetate after basal measurement. For mitochondrial stress and glycolysis experiments, cells were incubated with acetate for 24 h or received acetate during the experiment. Basal mitochondrial respiration in the presence of acetate (basal cellular respiration minus non-mitochondrial respiration), proton leak (oligomycin inhibited respiration minus non-mitochondrial respiration), ATP turnover-driven respiration (basal respiration minus oligomycin inhibited respiration), maximal mitochondrial respiratory capacity (uncoupler inhibited respiration) and non-mitochondrial respiration (rotenone-antimycin A inhibited respiration) were measured as OCR, as per manufacturer's instructions, by adding oligomycin (Sigma-Aldrick, UK, 1 μM) carbonyl cyanide 4-trifluoromethoxy phenylhydrazone (FCCP, Sigma-Aldrich, UK), HT29: 1 μM, HCT116: 0.5 μM) and rotenone and antimycine mix (Sigma-Aldrich, UK, 0.5 μM). Glycolysis, glycolytic capacity, and glycolytic reserve were measured as ECAR by adding glucose (Sigma, HT29: 4,500 mg/L, HCT116: 2,000 mg/L), oligomycin (using the doses mentioned above) and 2DG (50 mM, Sigma-Aldrich, UK). Metabolic potential was also measured at baseline and under stress by treating with oligomycin and FCCP simultaneously. For mitochondrial function, glycolysis and metabolic potential experiments, OCR and ECAR were normalized with total protein content at the end of the experiment quantified by Pierce™ BCA Protein Assay (ThermoFisher Scientific, USA).

### Measurement of ACSS1 and ACSS2 mRNA Expression and Protein Levels

To assess mRNA expression by quantitative RT-PCR, cells were seeded 5 × 10^6^ cells per flasks. Next day cells were treated with control or 10 mM acetate for 24 h. Cells were harvested, and total mRNA was extracted using RNeasy kit (Qiagen, USA) according to manufacturer's instructions. RT reactions were performed using SuperScript™ IV First-Strand Synthesis System (ThermoFisher Scientific, USA) using 1 μg/mL RNA concentration and used in RT-PCR. Quantitative PCR reactions were performed using TaqMan™ Universal Master Mix II, no UNG (ThermoFisher Scientific, USA) (*n* = 5) using Applied Biosystems 7500 Fast Real-Time PCR System (Life Technologies, USA). Taqman gene expression assays (ThermoFisher Scientific, USA) were used for β*-actin* (Hs01060665_g1), *ACSS1* (Hs00287264_m1), and *ACSS2* (Hs00218766_m1).

To assess protein levels of ACSS1 and ACSS2, cells were seeded 6 × 10^6^ cells per flask. The following day cells were treated with control or 10 mM acetate for 24 h (*n* = 3). Cells were collected and total protein was extracted after three freeze thaw cycles. ELISAs (MyBioSource, USA) were performed according to manufacturer's instructions and read at 450 nm using SPECTROStar Nano.

### Lipid Detection and Measurement

Cells were seeded in 6 well plates at a density of 500 × 10^3^ cells per well. After 24 h treatment with control or acetate (1 and 10 mM), cells were stained with Oil red O (ORO) (Sigma-Aldrich, UK) following the manufacturer's instructions. Staining was then extracted using 1 ml of isopropanol and measured at 510 nm in cuvettes using a JENWAY 6300 Spectrophotometer (Cole-Parmer, UK) (18).

### Statistical Analysis

All statistical analysis was performed using GraphPad Prism (GraphPad Software, USA). Data are presented as mean ± standard deviation (SD) or as mean ± standard error of mean (SEM). Statistical significance was calculated with Student's *t*-test or one-way ANOVA. Significance was accepted at the level of ^*^p < 0.05, ^**^p < 0.01, ^***^p < 0.001.

## Results

### Acetate Treatment Reduces Cell Viability and Proliferation

**Figure 1:**
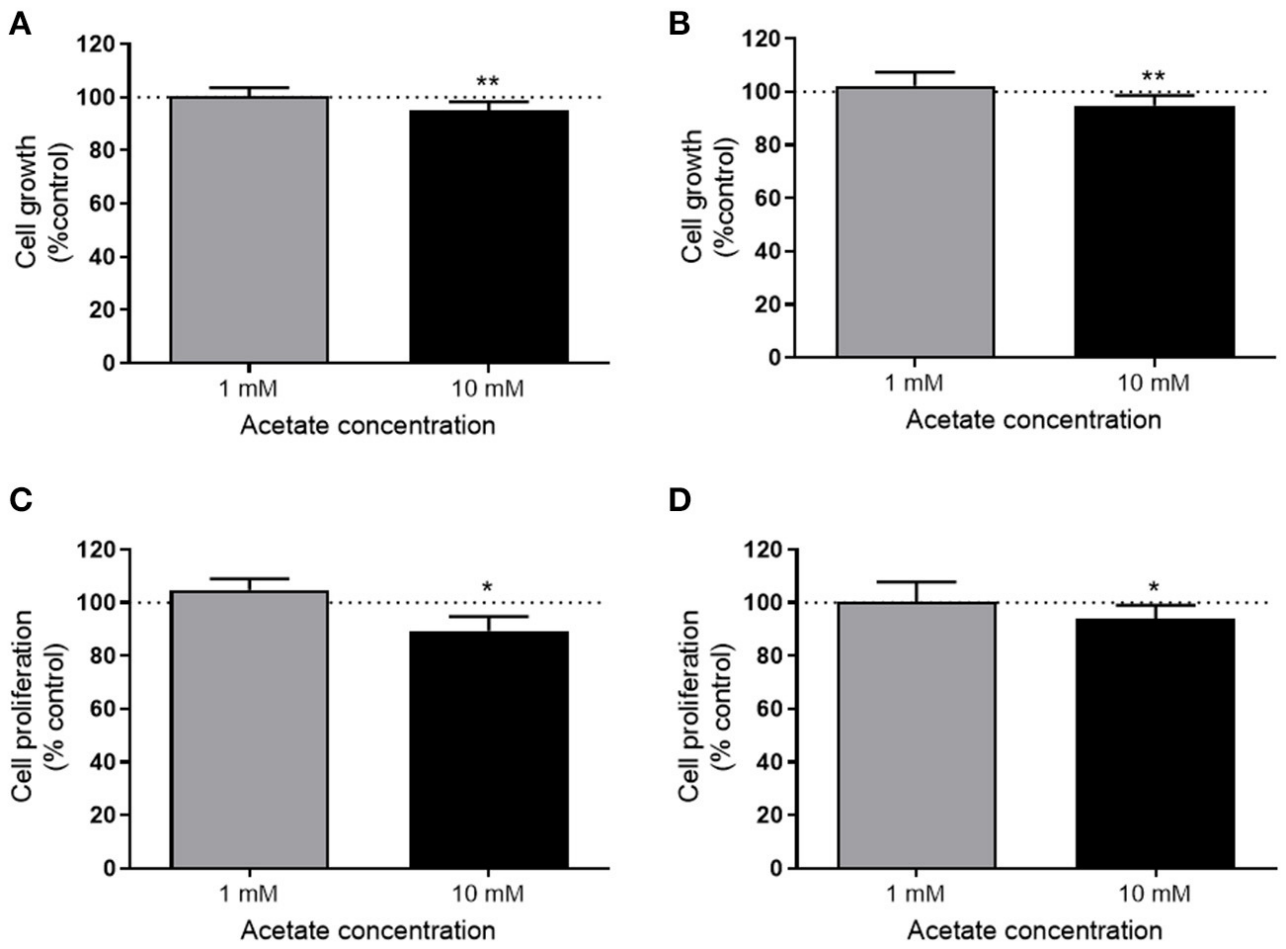
Acetate reduces proliferation. Percent change from control in growth (measured by SRB assay) of HT29 (*n* = 3) **(A)** and HCT116 (*n* = 4) **(B)** cell lines treated with 1 or 10 mM acetate for 24 h. Percent change of cell proliferation (measured by BRDU assay) from control of HT29 (*n* = 4) **(C)** and HCT116 (*n* = 6) **(D)** cell lines treated with 1 or 10 mM acetate for 24 h. All data are shown as mean ± SD, *p < 0.05 and **p < 0.01.

The viability of both HT29 and HCT116 cells was significantly reduced after 24 h treatment with 10 mM acetate (Figures 1A,B), however, there was no effect with 1 mM acetate treatment. A similar effect was observed on cell proliferation, with 10 mM acetate treatment reducing proliferation on both cell lines, whilst 1 mM acetate treatment had no significant effect (Figures 1C,D). No significant change was observed in ATP/ADP ratio of HT29 cells (Control: 9.24 ± 5.06, 1 mM: 12.6 ± 6.28, and 10 mM: 4.01 ± 1.44) or HCT116 cells (0: 6.32 ± 3.9, 1 mM: 8.5 ± 9.36, and 10 mM: 6.07 ± 0.97).

### Acetate Treatment Increases ROS Levels

**Figure 2:**
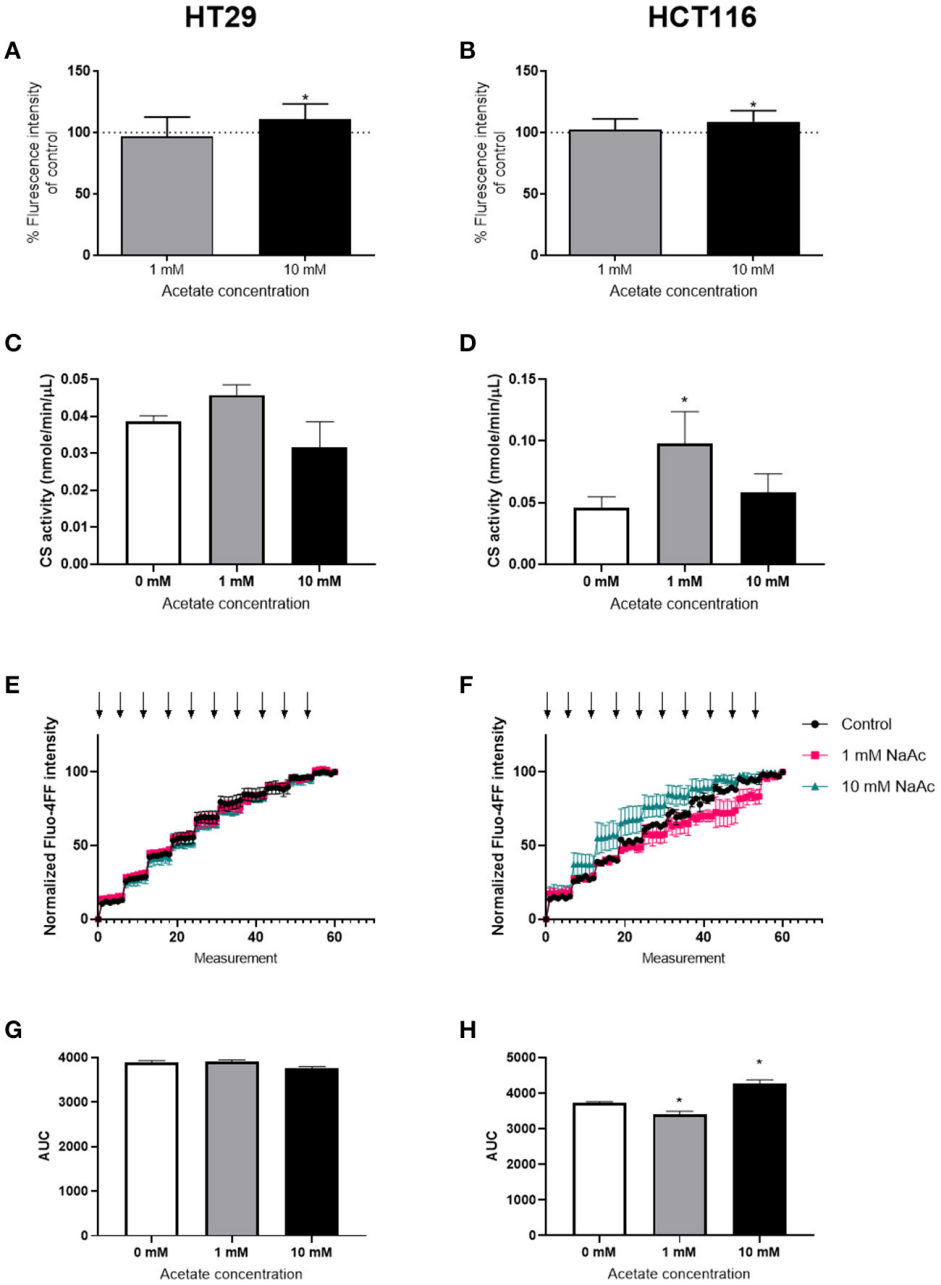
Acetate increases ROS production. Percent change from control of ROS produced by HT29 (*n* = 9) **(A)** and HCT116 (*n* = 9) **(B)** cell lines treated with 1 or 10 mM acetate for 24 h. Citrate synthase activity (nmole/min/μL) of HT29 **(C)** and HCT116 **(D)** cell lines (*n* = 3) treated with 1 or 10 mM acetate for 24 h. CRC curve (*n* = 3) shown as normalized fluorescent intensity of mitochondria isolated from HT29 **(E)** and HCT116 **(F)** cell lines treated with 1 and 10 mM acetate for 24 h. Each arrow represents CaCl_2_ injection. Area under the CRC curves of HT29 **(G)** and HCT116 **(H)** cell lines. All data are shown as mean ± SD except from **(E,F)**, which are shown as mean ± SEM, *p < 0.05.

ROS levels were significantly increased after 24 h treatment with 10 mM acetate in both HT29 (Figure 2A) and HCT116 cell lines (Figure 2B). However, treatment with 1 mM acetate did not alter ROS levels (Figures 2A,B), nor did a shorter 15 min treatment with either acetate concentration (data not shown).

Assessment of CS activity showed that acetate treatment at 10 mM caused no change in either cell lines. However, there was a significant increase in CS activity following treatment with 1 mM acetate in HCT116 cell line, suggesting increased mitochondrial density (Figures 2C,D).

The CRC assay showed a significant increase in HCT116 at 10 mM and a significant decrease at 1 mM suggesting opening of mPTP sooner and later than control, respectively. No change was observed in HT29 cell line (Figures 2E–H).

### Acetate Affects Cellular Bioenergetics

**Figure 3:**
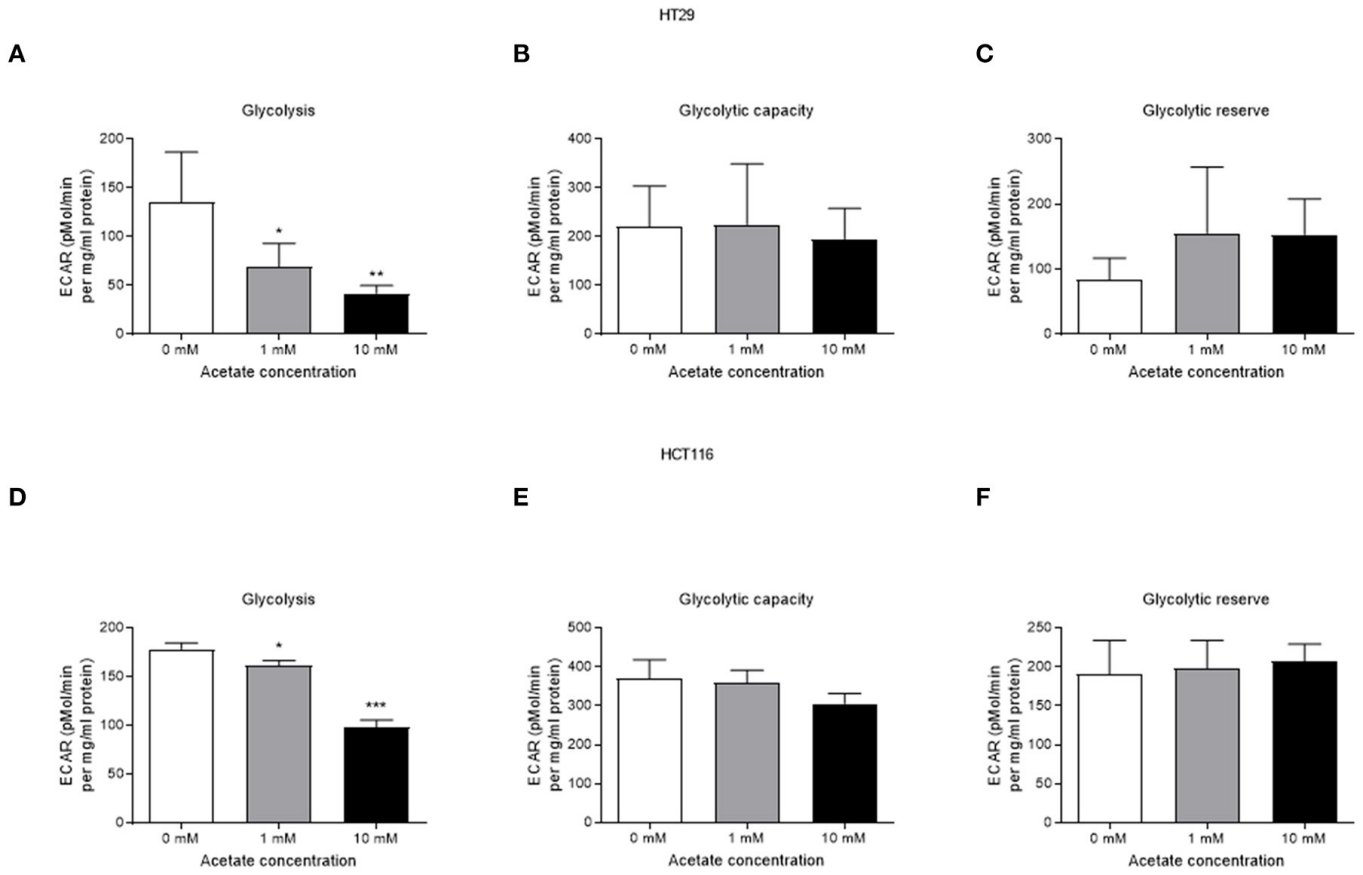
Acetate reduces glycolysis in a dose dependent manner. **(A)** glycolysis, **(B)** glycolytic capacity, and **(C)** glycolytic reserve of HT29 cell line treated with 1 and 10 mM acetate assessed by ECAR (a representative of lactate production) (*n* = 4), **(D)** glycolysis, **(E)** glycolytic capacity, and **(F)** glycolytic reserve of HCT116 cell line treated with 1 and 10 mM acetate assessed by ECAR (*n* = 3). All data are shown as mean ± SD, *p < 0.05, **p < 0.01, and ***p < 0.001.

Acute acetate treatment (1 and 10 mM), where acetate was given to cells during measurements, did not alter basal respiration, ATP production, proton leak, maximal respiration, non-mitochondrial respiration or spare respiration, in either cell line (Supplementary Figures 1, 2). Similarly, no effects were observed after 24 h incubation with acetate (Supplementary Figures 3, 4). Glycolysis was suppressed in a dose dependent manner in both HT29 (Figures 3A–C) and HCT116 (Figures 3D–F) cell lines following both 1 and 10 mM acute acetate treatment.

**Figure 4:**
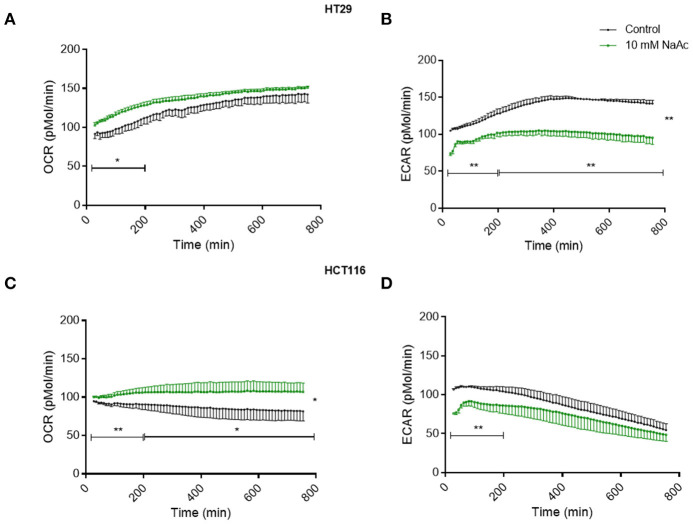
Acetate causes changes in metabolism (time-course study). Time-course measurement of OCR (pmol/min) and ECAR (pmol/min) for HT29 cell line **(A,B)**, respectively and HCT116 cell line **(C,D)**, respectively, treated with control and 10 mM acetate, measured continuously for 12 h, shown as change from baseline (OCR and ECAR measurements before acetate administration, *n* = 4 for HT29 and 3 for HCT116). All data are shown as mean ± SEM. Data analyzed by AUC and student's *t*-test, *p < 0.05 and **p < 0.01.

Time-course study of cells treated with 10 mM acetate showed an increased oxygen consumption rate (OCR) accompanied by a reduced extracellular acidification rate (ECAR) in HT29 (Figures 4A,B) and HCT116 cell lines (Figures 4C,D). OCR of HT29 cell line was significantly increased for the first 200 min but not thereafter (Figure 4A) whereas ECAR was significantly reduced throughout the treatment (Figure 4B). For HCT116 cell line, OCR of acetate treated cells was significantly higher throughout the experiment (Figure 4C) whilst ECAR was significantly reduced during the first 200 min of treatment (Figure 4D).

### Acetate Affects *ACSS1/2* Expression

**Figure 5:**
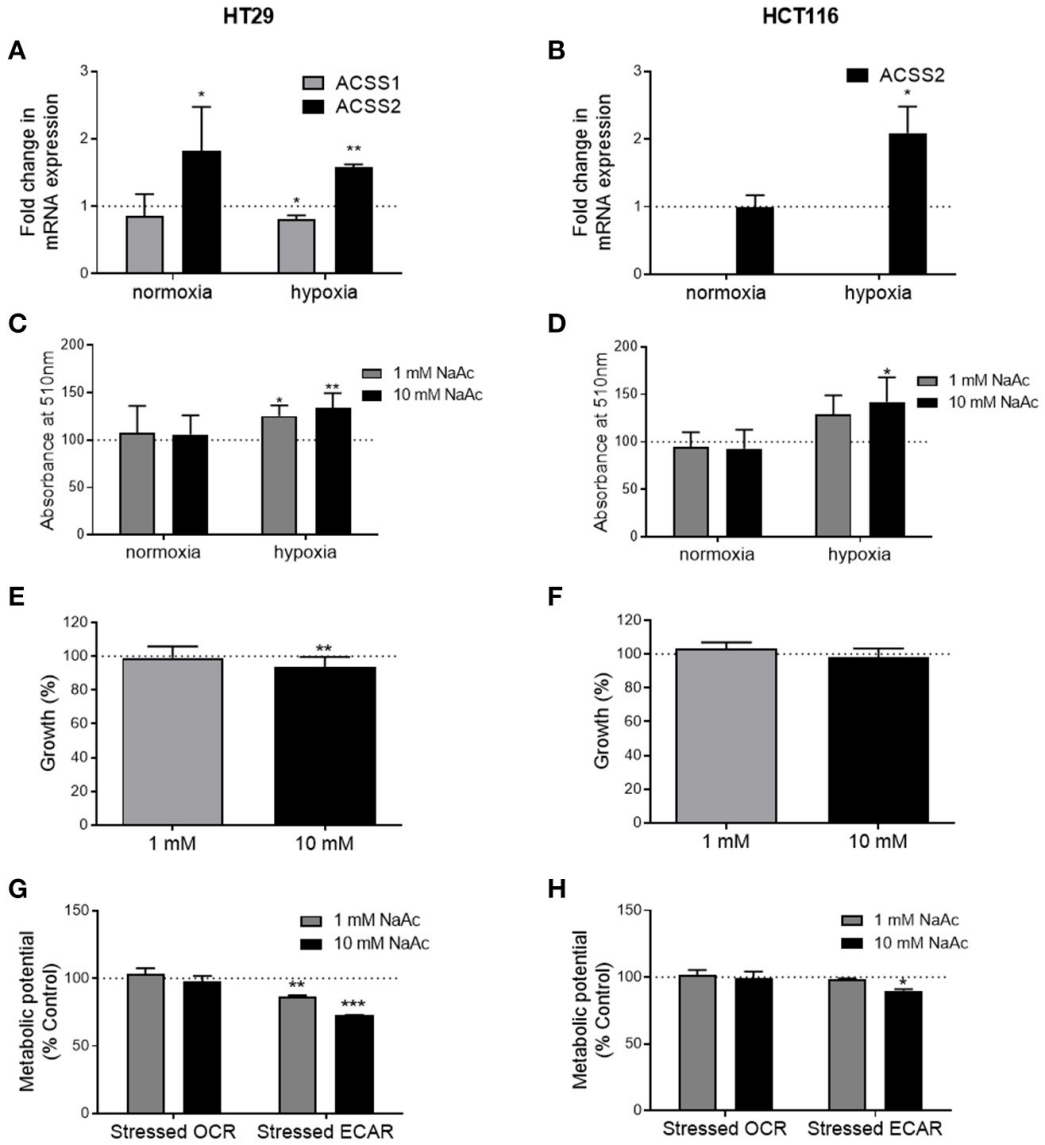
Effects of acetate under metabolic stress are modified. **(A)** Fold change in *ACSS1* and *ACSS2* mRNA expression of 10 mM acetate treated HT29 cells under normoxia and hypoxia compared to control treated cells (represented by dotted line, *n* = 3), **(B)** fold change in *ACSS1* and *ACSS2* mRNA expression of 10 mM acetate treated HCT116 cells under normoxia and hypoxia compared to control treated cells (represented by dotted line, *n* = 3), **(C)** relative change in ORO staining, measured as absorbance at 510 nm of HT29 cell line treated with 10 mM acetate from control (represented by dotted line, *n* = 5). **(D)** Relative change in ORO staining, measured as absorbance at 510 nm of HCT116 cell line treated with 10 mM acetate from control (represented by dotted line, *n* = 5). Percent change from control in growth of HT29 (*n* = 3) **(E)** and HCT116 (*n* = 3) **(F)** cell lines treated with 1 or 10 mM acetate for 24 h under hypoxia, metabolic potential of HT29 (*n* = 3) **(G)** and HCT116 (*n* = 5) **(H)** cell lines treated with 1 and 10 mM acetate measured as stressed OCR and ECAR under stressed conditions induced by simultaneous treatment with oligomycin and FCCP. All data represented as mean ± SD, *p < 0.05, **p < 0.01, and ***p < 0.001.

Under normal oxygen conditions, acetate increased the expression of *ACSS2* in the HT29 cell line but not *ACSS1* (Figure 5A). In the HCT116 cell line, expression of both enzymes remained unaffected (Figure 5B). Under hypoxia, *ACSS1* expression was suppressed in HT29 cell line while *ACSS2* expression was increased in both cell lines (Figures 5A,B). No change was observed in protein levels of *ACSS1* and *ACCS2* in either cell lines under normoxia (Supplementary Figure 5).

### Acetate Increases Lipid Deposition Under Hypoxia

ORO staining showed increased lipid levels following 24 h 10 mM acetate treatment under hypoxic conditions in both cell lines. One millimolar treatment also increased ORO staining in HT29 cell line but did not reach significance in HCT166 cell line. No effect was observed under normal oxygen conditions (Figures 5C,D).

### Effect of Acetate on Cell Growth Under Hypoxia

Under hypoxic conditions, 24 h acetate (10 mM) treatment reduced proliferation of HT29 cell line but had no effect on HCT116 cells (Figures 5E,F).

### Effect of Acetate on Metabolic Potential Under Drug Induced Metabolic Stress

Under stressed conditions both 1 and 10 mM acute acetate treatment reduced the metabolic potential of the ECAR, which indicates the preferred energy pathway of the HT29 cells (Figure 5G). 10 mM acute acetate treatment reduced the metabolic potential of ECAR in HCT116 cells, with no effect observed at the lower acetate concentration (Figure 5H).

## Discussion

In this study we report that acetate increases oxygen consumption and ROS production while reducing glycolysis and cell proliferation. We showed that these effects occurred in the absence of suppressed *ACSS2* expression. Under conditions of metabolic stress, in this case hypoxia, acetate's effect on the HT29 cell line was maintained but no longer observed in the HCT116 cell line.

Under normal metabolic conditions, cellular acetate is metabolized into acetyl-CoA by two different enzymes, mitochondrial *ACSS1* (19) and cytosolic *ACSS2* (20). The ultimate fate of acetate is thought to depend on the enzyme responsible for converting it to acetyl-CoA. Acetyl-CoA produced by *ACSS1* is predominantly used for oxidation (20) whilst acetyl-CoA produced by *ACSS2* is destined for lipid synthesis (19). Previous work has shown that tumor growth is reduced by suppressing *ACSS2* expression in most cancers (12, 13). Cells are more dependent on acetate metabolism when metabolically stressed, including hypoxia and/or nutrients (13), which may explain increased *ACSS2* expression in cancer. Indeed, Mashimo et al. reported that *ACSS2* expression is increased in brain tumors (11). Although most work has focused on *ACSS2*, Lakhter et al. showed that suppression of *ACSS1* expression results in reduced cell growth, accompanied by increased OXPHOS following acetate treatment (21). Hence, it appears that under some circumstances, endogenous acetate could play a role in cancer progression (12, 13, 21).

Despite this, several studies have shown that acetate can reduce cell growth in stem-like cells and colon cancer (2, 10, 22, 23). Otto Warburg was first to show that cancer cells preferentially display aerobic glycolysis and their growth can be modified by respiration (24), a key hallmark of cancer (25). Schulz et al. demonstrated that growth and colony formation of several colon cancer cell lines can be reduced by reversing the Warburg effect through increasing mitochondrial oxygen consumption (26, 27). We have previously shown that while acetate can enhance the mitochondrial function of non-cancerous cells (14), it can also reduce tumor sizes in xenograph models of colon cancer (1). We therefore hypothesized that the impact of acetate on mitochondrial function could be the underlying mechanism. Although the effect on cell proliferation observed was small, this was in response to acute acetate treatment. Our previous study showed that chronic acetate treatment reduces tumor volume by half (1). Under normoxia, we found no effect at zero or 24 h in mitochondrial function but continuous measurements showed that acetate treatment increased oxygen consumption and reduced glycolysis in both HT29 and HCT116 cells lines, reversing the Warburg effect. Pavlides et al. suggested that the Warburg effect is not limited to cancer cells and stromal cells can switch metabolism to aerobic glycolysis, termed “Reverse Warburg Effect” (28) and that inhibition of glycolysis reduces tumor growth (29). As we observed reduced glycolysis following acetate treatment, it may be worthwhile for further studies to explore whether acetate can be used to reverse the “Reverse Warburg Effect.”

As expected, changes in oxygen consumption and glycolysis were accompanied by increased ROS production, as glycolysis has been shown to be a ROS scavenger (30). Cancer cells are heavily dependent on glycolysis for energy production, as well as generating the macromolecules needed for proliferation and intermediates for redox homeostasis (30). By reducing glycolysis, in our case with exogenous acetate, ROS production is increased thus providing sufficient stress to explain the reduction in tumor growth (31). ROS may cause cell death through the opening of mPTP, which triggers the release of more ROS, which in turn becomes detrimental to mitochondria and the cell (32). We have observed some evidence that acetate causes a biphasic effect on mPTP opening in HCT116 cell line, a lower dose delays its opening, while the higher dose accelerates it. This effect was not observed in HT29 cell line and warrants further exploration to ascertain if chronic treatments or higher doses of acetate would induce an effect.

In the current study, *ACSS2* expression, intracellular lipid deposition and cellular proliferation, in the presence of acetate, were clearly dependent on environmental conditions. Under normoxia, cell proliferation was reduced independent of *ACSS2* expression, however under hypoxia both ACSS2 expression and lipid levels were increased with proliferation reduced only in the HT29 cell line. *ACSS2* is involved in synthesis of lipids and growth of breast cancer cells in hypoxia (13). The importance of lipid metabolism in tumor growth has previously been demonstrated as the blocking of lipid synthesis protects against tumor growth (33) and cancer cells can reduce oxidative stress and cell death by upregulating lipogenesis (34). Gao et al. showed that *ACSS1* and *ACSS2* are involved in activation of genes involved in lipogenesis through increased histone acetylation under hypoxia (35). Our results suggest that the HCT116 cell line is more glycolytic than the HT29 cell line. As the cells increase glycolysis under hypoxic conditions (36), any suppression that acetate caused may not have been of sufficient magnitude to reduce cell growth in the HCT116 cell line. Therefore, we observed a reduction in cell proliferation under normal oxygen conditions independent of *ACSS2* expression while under hypoxic conditions, *ACSS2* expression could protect against cell death caused by acetate depending on the glycolytic rate of the cells. Another mechanism by which acetate increases proliferation under hypoxia is through acetylation and activation of Hypoxia Inducible Factor-2 (*HIF-2*) in response to stressors; knocking down *ACSS2* in hypoxia reduces cell proliferation (37). Acetate supplementation has been shown to increase cell proliferation both *in vivo* and *in vitro* through *ACSS2* and *HIF-2* pathway in a fibrosarcoma cell line, where *ACSS2* translocates into nucleus and acetyl-CoA increases *HIF-2* acetylation thereby increasing growth and proliferation (38). This contradictory finding to our present study may relate to differences in metabolism within the different cell lines used, as well as the dose of acetate used (5 mM).

**Figure 6:**
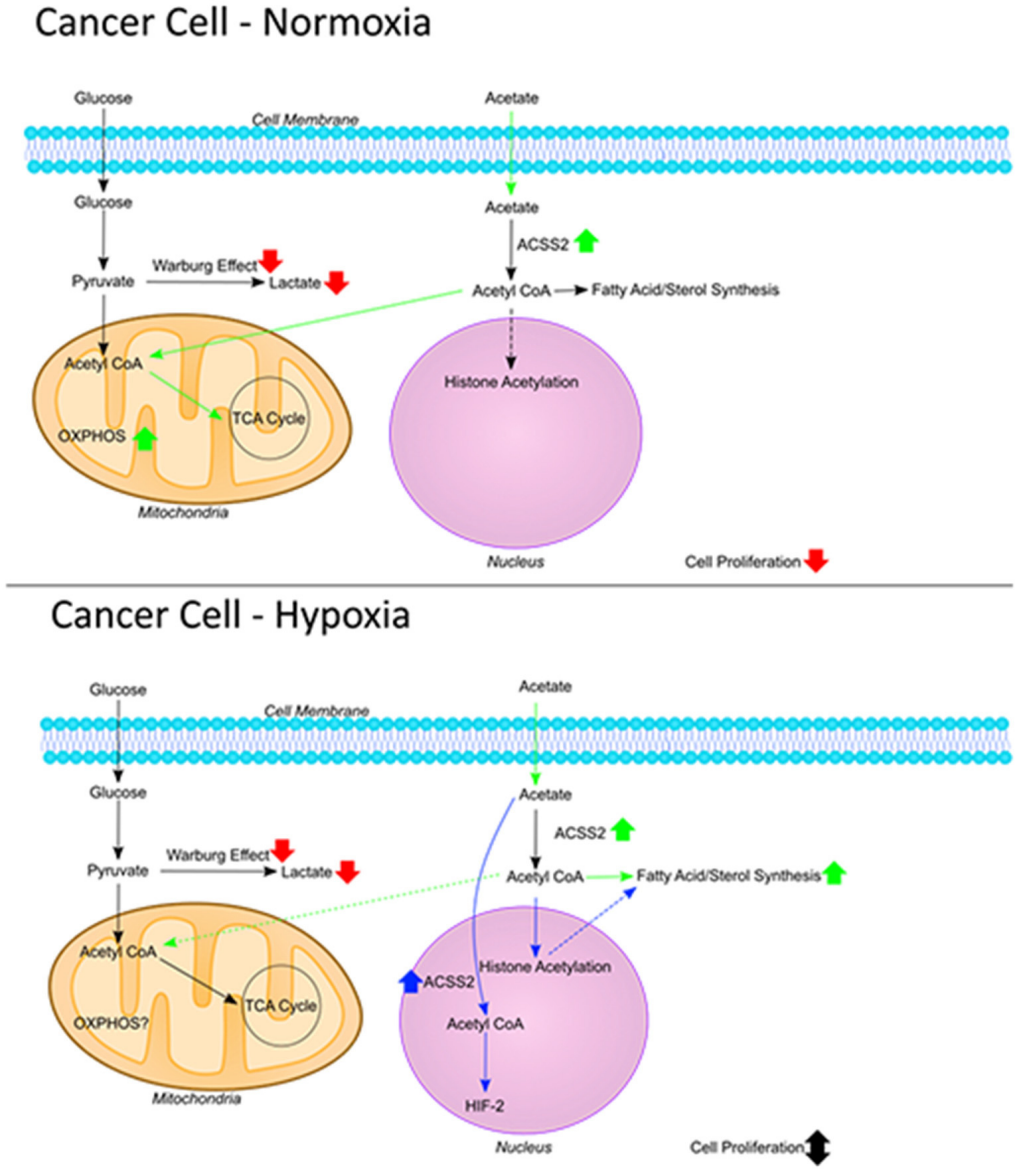
The fate of acetate. Figure summarizing the fate of exogenous acetate in cancer cells under normoxia and hypoxia. In non-cancerous cells, exogenous acetate is taken into mitochondria and increases the rate of TCA cycle and OXPHOS. In cancer cells, mitochondrial function is decreased and lactate production is increased (Warburg effect) so endogenous acetate ends up being made into lipids and used in histone acetylation which can increase cell proliferation. Under normoxic conditions in cancer cells, exogenous acetate increases OXPHOS, reducing glycolysis which results in increased ROS and reduced proliferation. Under hypoxia in cancer cells, exogenous acetate increases lipid synthesis, or it can increase HIF-2 signaling [shown by Chen et al. (38)] both of which can protect the cells from cell death induced by reduced glycolysis.

These results, taken together with findings from previous studies, suggest two opposing functions for endogenous and exogenous acetate. Endogenous acetate is vital for cancer cell proliferation as shown by reduced cell growth in cells where *ACSS1/2* is silenced (11–13, 21), whereas exogenous acetate reduces cell proliferation. This was shown previously (1) and confirmed by the current work where reduced cell proliferation was observed despite increased *ACSS2* gene expression (Figure 6).

Although we observed significant changes in cellular bioenergetics which can explain the anti-proliferative effects of acetate, acetate has also been shown to be involved in modifying histone acetylation. In addition to its effects on lipid synthesis and HIF-2 activation (36–38), dietary acetate supplementation in rats was shown to reduce HDAC activity and increase histone acetylation in the brain (39, 40). We have also previously shown that chronic treatment with acetate can reduce expression of various HDACs (1). Here, we show that epigenetic modifications by acetate, such as changes in expression of *ACSS2*, alone is not enough in explaining its effects and bioenergetic modifications by acetate can help us understand how it reduces proliferation. Mitochondrial proteins are highly acetylated [see review by Baeza et al. (41)] and as cellular energy status modulates mitochondrial protein acetylation [see review by Anderson and Hirschey (42)], acetate can also play a role in acetylation of mitochondrial proteins. Nevertheless, studies combining genetic, epigenetic, and metabolic measurements will be necessary to fully understand the effects of acetate.

In summary, we have shown that exogenous acetate can reduce cell proliferation through modulation of mitochondrial function. It increases OXPHOS, reduces glycolysis and increases ROS which we believe is the mechanism underlying its actions. The effect on cell growth however, depends on the cell line and factors, such as glycolytic rate of the cell and metabolic stressors, such as hypoxia.

## Data Availability Statement

The raw data supporting the conclusions of this article will be made available by the authors, without undue reservation.

## Author Contributions

MS-A designed and performed the experiments, performed the data analyses, and drafted the manuscript. RM and NS performed the continuous cellular bioenergetics and cell proliferation experiments, respectively. All authors contributed to the article and approved the submitted version.

## Conflict of Interest

The authors declare that the research was conducted in the absence of any commercial or financial relationships that could be construed as a potential conflict of interest.
