# Supplementary material for: Acetate Induces Growth Arrest in Colon Cancer Cells Through Modulation of Mitochondrial Function
Source: Front Nutr. 2021 Apr 15;8:588466. doi: 10.3389/fnut.2021.588466 (PMC8081909; doi:10.3389/fnut.2021.588466)
Supplement: Supplementary file 1 [file Data_Sheet_1.docx]

Supplementary Material

## Supplementary Figures


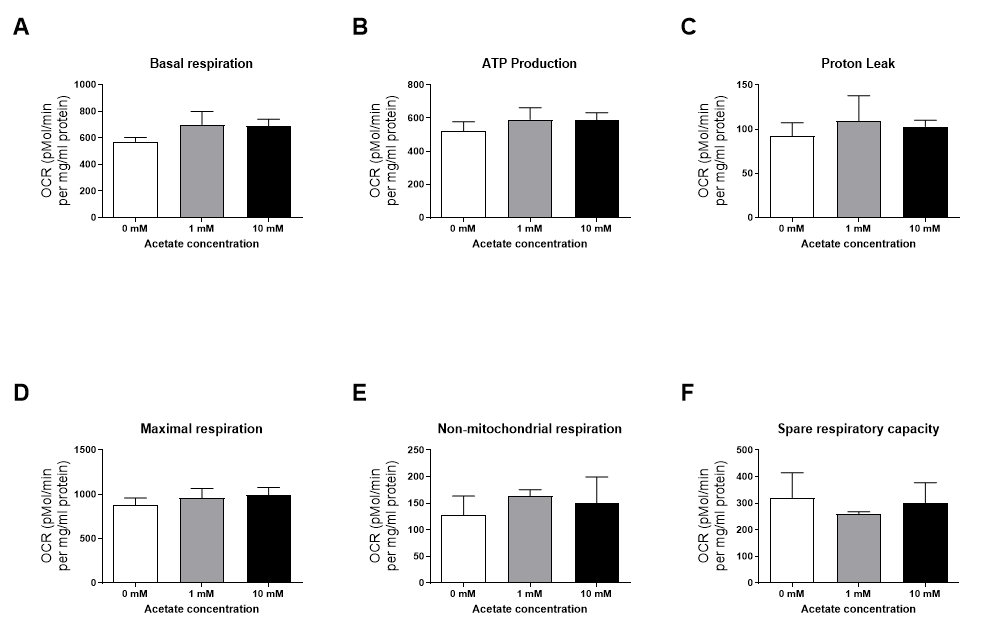


**Figure S1.** Acute effect of acetate on mitochondrial bioenergetics of HT29 cell line. Mitochondrial function, assessed by OCR, (pmol/min per mg/ml of protein) basal respiration **(A)**, ATP production **(B)**, proton leak **(C)**, maximal respiration **(D)**, non-mitochondrial respiration **(E)**, spare respiratory capacity **(F)** of HT29 cell line treated with 1 and 10mM acetate measured immediately after treatment (n=3). All data are shown as mean ± SD.


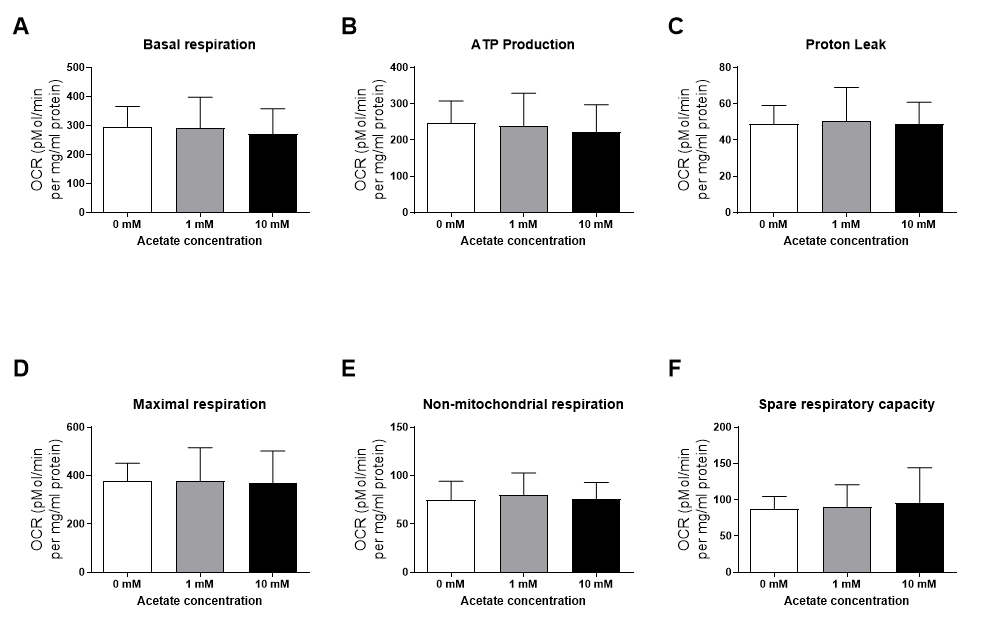


**Figure S2.** Acute effect of acetate on mitochondrial bioenergetics of HCT116 cell line. Mitochondrial function, assessed by OCR, (pmol/min per mg/ml of protein) basal respiration **(A)**, ATP production **(B)**, proton leak **(C)**, maximal respiration **(D)**, non-mitochondrial respiration **(E)**, spare respiratory capacity **(F)** of HCT116 cell line treated with 1 and 10mM acetate measured immediately after treatment (n=5). All data are shown as mean ± SD.


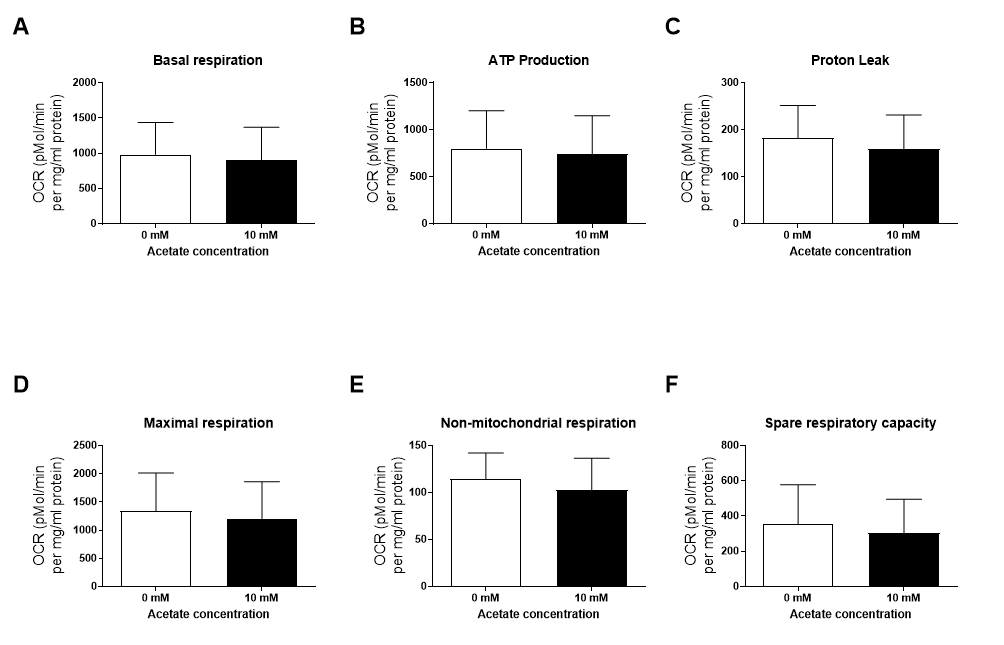


**Figure S3.** Effect of 24 h acetate treatment on mitochondrial bioenergetics of HT29 cell line. Mitochondrial function, assessed by OCR, (pmol/min per mg/ml of protein) basal **(A)**, ATP production **(B)**, proton leak **(C)**, maximal respiration **(D)**, non-mitochondrial respiration **(E)**, spare respiratory capacity **(F)** of HT29 cell line treated with 10mM acetate measured 24 h after treatment (n=3). All data are shown as mean ± SD.


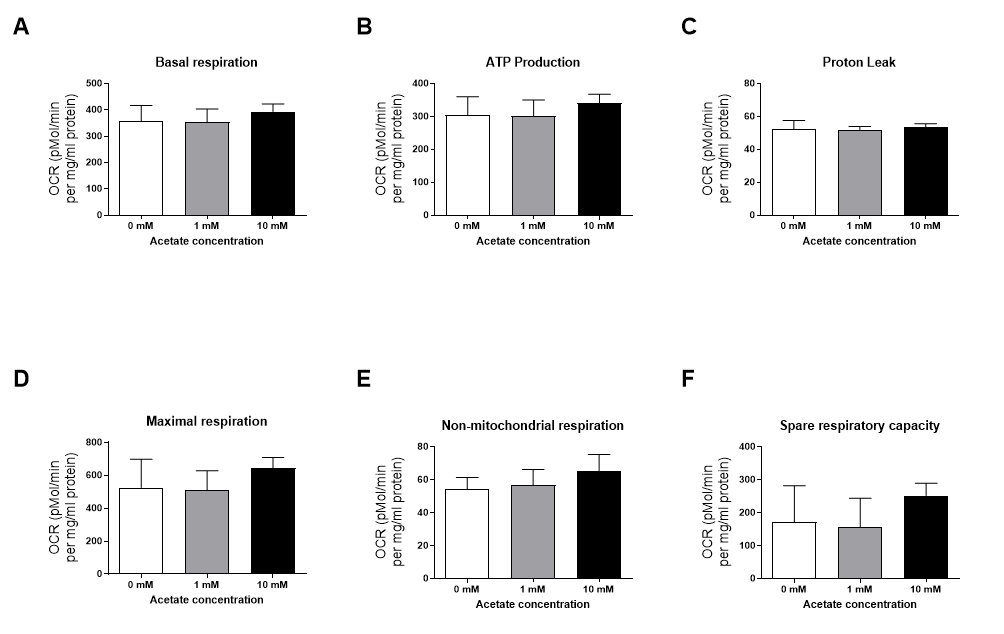


**Figure S4.** Effect of 24 h acetate treatment on mitochondrial bioenergetics of HCT116 cell line. Mitochondrial function, assessed by OCR, (pmol/min per mg/ml of protein) basal respiration **(A)**, ATP production **(B)**, proton leak **(C)**, maximal respiration **(D)**, non-mitochondrial respiration **(E)**, spare respiratory capacity **(F)** of HCT116 cell line treated with 1 and 10mM acetate measured 24 h after treatment (n=4). All data are shown as mean ± SD.


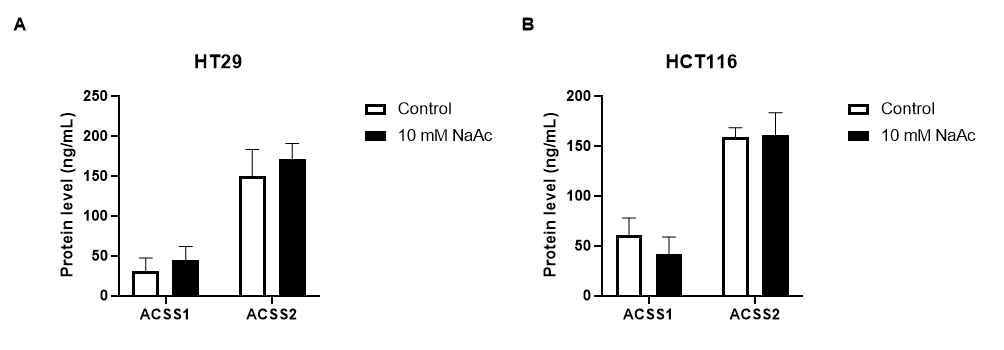


**Figure S5.** Effect of acetate on ACSS1 and ACSS2 protein levels. ACSS1 and ACSS2 protein levels (ng/mL) after 24 h acetate treatment (n=3) for HT29 **(A)**, and HCT116 **(B)** cell lines. All data are shown as mean ± SD.
